# Pemphigoid associated with quinolone antibiotics: a real-world pharmacovigilance study of the FDA adverse event reporting system and literature-based evidence

**DOI:** 10.3389/fimmu.2026.1808910

**Published:** 2026-04-15

**Authors:** Xin Li, Danna Zhang, Jiangxin Xu, Zhirou Xu, Kan Xu, Junjie Cheng

**Affiliations:** 1 Department of Geriatrics, Integrated Traditional Chinese and Western Medicine Hospital of Zhejiang Province, Hangzhou, China; 2 Department of Pharmacy, Integrated Traditional Chinese and Western Medicine Hospital of Zhejiang Province, Hangzhou, China; 3 Jiangsu Key Laboratory of Drug Discovery and Translational Research for Brain Diseases and College of Pharmaceutical Sciences, Soochow University, Suzhou, Jiangsu, China

**Keywords:** FAERS, immune-related adverse drug reactions, pemphigoid, pharmacovigilance, quinolone antibiotics

## Abstract

**Objective:**

Pemphigoid is a rare autoimmune disorder that may be drug-induced. This study aims to investigate the potential association between pemphigoid and quinolone using the FDA Adverse Event Reporting System (FAERS) database.

**Methods:**

FAERS reports were analyzed using disproportionality methods, including the reporting odds ratio (ROR), proportional reporting ratio (PRR), and Bayesian confidence propagation neural network (BCPNN). Subgroup analyses were performed by age, sex, and reporter type. An interaction analysis integrating published literature on quinolone-associated pemphigoid (QAP) was conducted to assess consistency in reporting disproportionality, demographics, latency, and clinical outcomes.

**Results:**

A total of 183 reports of quinolone-associated pemphigoid were identified. Ofloxacin accounted for the largest proportion of cases (47.0%), followed by ciprofloxacin (26.2%) and levofloxacin (23.0%). Significant disproportionality signals were detected for ofloxacin (ROR = 2.75), ciprofloxacin (ROR = 2.31), levofloxacin (ROR = 2.53). A remarkably high signal was also noted for norfloxacin (ROR = 25.14); however, this requires extremely cautious interpretation due to the very small number of cases (n=3). Most pemphigoid events occurred within 30 days of quinolone initiation, indicating a short latency pattern consistent with immune-mediated blistering reactions. Subgroup analyses revealed differential reporting patterns by age and sex. Furthermore, the interaction analysis with published literature corroborated the FAERS findings, demonstrating consistent patterns in demographic susceptibility (predominantly elderly and female), short latency, and clinical outcomes.

**Conclusions:**

This study demonstrates that several quinolone antibiotics are associated with an increased reporting frequency of pemphigoid in FAERS. The early onset and consistency across data sources support quinolone-associated pemphigoid as a clinically meaningful immune-related adverse drug reaction, underscoring the importance of early clinical recognition and monitoring during the initial phase of quinolone therapy.

## 1 Introduction

Pemphigoid is a rare but potentially life-threatening autoimmune skin disease, primarily characterized by tense blisters and erosions on the skin or mucous membranes (1–3). Pemphigoid diseases include the common Bullous pemphigoid (BP) as well as Mucous membrane pemphigoid (MMP), Linear IgA bullous dermatosis (LABD), and Epidermolysis acquired bullous (EBA) (4). This group of disorders occurs more frequently in the elderly, and its incidence has been increasing in recent years (5–8). Pemphigoid is associated with various infections, autoimmunity, cardiovascular issues, and neuropsychiatric comorbidities (9–11). Numerous medications have been implicated in triggering pemphigoid, including aldosterone antagonists, DPP-4 inhibitors, Nivolumab, Nemolizumab, and neuropsychiatric medications (12–17). Certain drugs, such as dipeptidyl peptidase 4 inhibitors, aspirin, and durvalumab, can induce antibody production by acting as haptens and binding to proteins in the basement membrane, leading to pemphigoid. Other agents, such as PD-1 inhibitors and ipilimumab, may activate the complement system or stimulate autoimmune responses to induce pemphigoid (18–20). Therefore, a systematic evaluation of drug-induced effects in patients with pemphigoid is crucial for enhancing medication safety and improving treatment outcomes.

Quinolones are broad-spectrum antimicrobial agents that primarily exert bactericidal effects by inhibiting bacterial DNA gyrase and topoisomerase IV (21, 22). Due to their favorable oral bioavailability and tissue penetration, quinolones are widely used to treat respiratory, urinary tract, and skin or soft tissue infections (22). In addition to common adverse effects such as gastrointestinal symptoms and neurotoxicity, growing evidence indicates that quinolones may trigger severe autoimmune blistering disorders (23–25). However, comprehensive studies on the role of quinolones in the pathogenesis of pemphigoid remain limited.

The FDA Adverse Event Reporting System (FAERS) is one of the world’s largest pharmacovigilance databases, supporting the systematic collection, management, and analysis of reports related to potential adverse drug reactions to inform clinical decision-making (26–28). This study uses FAERS data to investigate pemphigoid adverse events (AEs) associated with quinolone antibiotics and pemphigoid. The findings will facilitate a therapeutic risk-benefit assessment of antimicrobial therapy, optimize pharmacovigilance practices, and improve patient outcomes by enhancing drug safety protocols.

## 2 Methods

### 2.1 Data sources and study design

**Figure 1:**
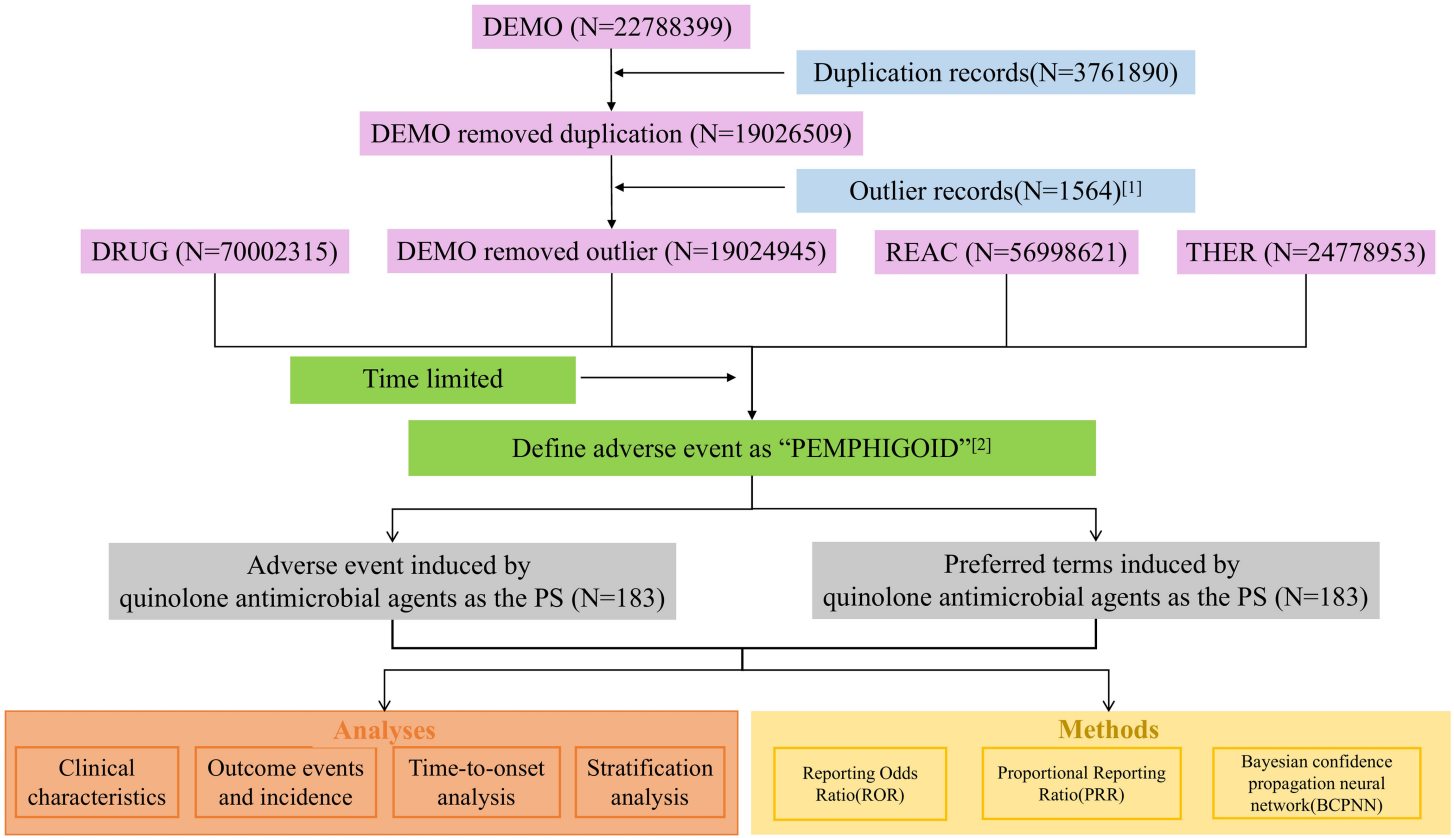
Technical roadmap. Illustrated is the initial dataset (DEMO) after exclusion of duplicate and outlier records, focusing on adverse events defined as”Pemphigoid” induced by quinolone antimicrobial agents as the primary suspect. Key metrics encompass the reporting odds ratio (ROR), proportional reporting ratio (PRR), Bayesian confidence propagation neural network (BCPNN) analysis, clinical characteristics, outcome events with their incidence, and time-to-onset. DEMO, Demographic and Administrative Information; DRUG, Drug Information; REAC, Reaction Information; THER, Therapy Dates and Timing.

Adverse events (AEs) reports were retrieved from the FDA Adverse Event Reporting System (FAERS,https://fis.fda.gov/extensions/FPD-QDE-FAERS/FPD-QDE-FAERS.html) covering Q1 2004-Q1 2025 (29, 30). Duplicate reports were removed per FDA-recommended criteria: using PRIMARYID, CASEID, and FDA_DT from the DEMO component, reports were sorted by these fields, with only the record with the largest PRIMARYID retained for duplicates. AE terms were standardized via MedDRA (v27.1) covering SOC to LLT hierarchies. Drug names were standardized with the WHO Drug Dictionary (2024 Q3), and approval timelines of 8 quinolones were obtained from the FDA Approved Drug Products Portal to define drug-specific reporting periods. After excluding 3 quinolones with inconsistent generic names or no pemphigoid-related Preferred Terms (PTs), 183 reports involving 5 quinolones as primary suspected drugs were included (**Figure 1**). All analyses were conducted using R software (v4.4.1).

Published studies on quinolone-induced pemphigoid were systematically retrieved from PubMed, Embase, and CNKI (search terms: “quinolone”, “ciprofloxacin”, “levofloxacin”, “ofloxacin”, “pemphigoid”, “drug-induced”). Inclusion criteria: (1) Full-text available; (2) Clear diagnosis of pemphigoid (pathological or immunofluorescence confirmation); (3) Definite quinolone exposure as the suspected cause; (4) Key information including patient age, gender, drug type, latency, and clinical outcome available. Finally, 9 eligible studies were included, covering 9 QAP cases (**Supplementary Table 1**). As quinolone-induced pemphigoid is a rare adverse event, only a limited number of case reports have been published to date. To ensure diagnostic accuracy and clear causal assessment in this study, we exclusively included individual cases confirmed by histopathology or direct immunofluorescence with a definitive temporal relationship to drug exposure. Consequently, the final sample size was relatively small.

### 2.2 Descriptive analysis

Demographic and clinical characteristics (Sex, Age, Weight, Reporting Year, Reporter’s Occupation/Continent, Outcome) were summarized. Missing values were labeled “NS”. Age was stratified into three groups: 18-44, 45-64, and >65 years; Weight into <80, and ≥80 kg. Reporters were categorized as Consumer (CN), Physician (MD), Pharmacist (PH), or Other (OT); Outcomes as Hospitalization (HO), Death (DE), or Other (OT, including recovery and symptomatic relief). For literature data, core variables including drug type, age, gender, latency, clinical manifestations, and treatment outcomes were extracted and standardized for interaction analysis.

### 2.3 Disproportionality analysis

We employed descriptive statistics to characterize the profiles of AE reports associated with five quinolones. Disproportionality analysis was performed using 2×2 contingency tables (**Table 1**) to compare observed versus expected frequency ratios between drug-exposed and unexposed cohorts, where the unexposed cohort comprised all other reports in the FAERS database for the same time period that did not list the target quinolone as a primary suspected drug.

**Table 1:** Fourfold table of proportional imbalance method.

Item	Target adverse events	All other adverse events	Total
Target drugs	a	b	a+b
All other drugs	c	d	c+d
Total	a+c	b+d	a+b+c+d

ROR = (a/b)/(c/d),95%CI = e^(ln(ROR) ± 1.96√(1/a + 1/b + 1/c + 1/d)).

PRR = (a/(a+b))/(c/(c+d)),95%CI = e^(ln(PRR) ± 1.96√(1/a + 1/(a+b) + 1/c + 1/(c+d)))

IC = log2(a(a+b+c+d)/((a + b)(a + c))),IC025= E(IC)-2√(V(IC))

To identify potential safety signals while balancing sensitivity and specificity—particularly for detecting immune-related adverse drug reactions within a spontaneous reporting system—we employed three complementary disproportionality metrics were applied to identify potential safety signals: the Reporting Odds Ratio (ROR, primary method), the Proportional Reporting Ratio (PRR), and the Bayesian Confidence Propagation Neural Network (BCPNN) (31, 32). Positive signals were defined independently for each metric: for ROR, as having ≥3 cases (a) and a lower limit of the 95% confidence interval >1 [ROR = (a/b)/(c/d)]; for PRR, as having ≥3 cases, a PRR value ≥2, and a χ² ≥4 [PRR = (a/(a+b))/(c/(c+d))]; and for BCPNN, as an information component lower bound (IC025) >0. A drug-event combination was flagged as a potential safety concern if it met the criteria for any one of these three metrics. We primarily interpreted and ranked signal strength based on ROR values, with PRR and BCPNN serving as supplementary validation. All analyses were conducted using R software (version 4.4.1). To assess the robustness of signals and explore potential effect modifiers, sensitivity analyses were conducted through subgroup stratification by age, sex, and reporter type.

### 2.4 Interaction analysis

To contextualize the pharmacovigilance signals within the broader scientific evidence and assess their external validity, a comparative analysis was conducted between the FAERS findings and relevant published literature. This interaction analysis aimed to use the literature as an auxiliary reference to explore clinical features (e.g., short latency, specific age/sex susceptibility, and clinical severity profile) observed in the FAERS data. The analysis evaluated the consistency of four key dimensions: the specific quinolones implicated and their relative risk signals (e.g., ROR values); the demographic patterns of reported cases, including age and sex distribution; the latency period from drug initiation to pemphigoid onset; and the clinical outcome profiles, particularly the proportion of severe outcomes such as hospitalization and death. The aim was to determine the coherence between the spontaneous reporting patterns in FAERS and the clinical-epidemiological characteristics documented in prior studies.

## 3 Results

### 3.1 Case selection

A total of 19,024,945 AE reports in the FAERS database from Q1–2004 to Q1–2025 were reviewed. The analysis was limited to reports based on the approval timelines of quinolones and focused on pemphigoid reactions. This process resulted in the identification of 183 reports involving five quinolones as PS drugs.

### 3.2 AE report overview

**Figure 2:**
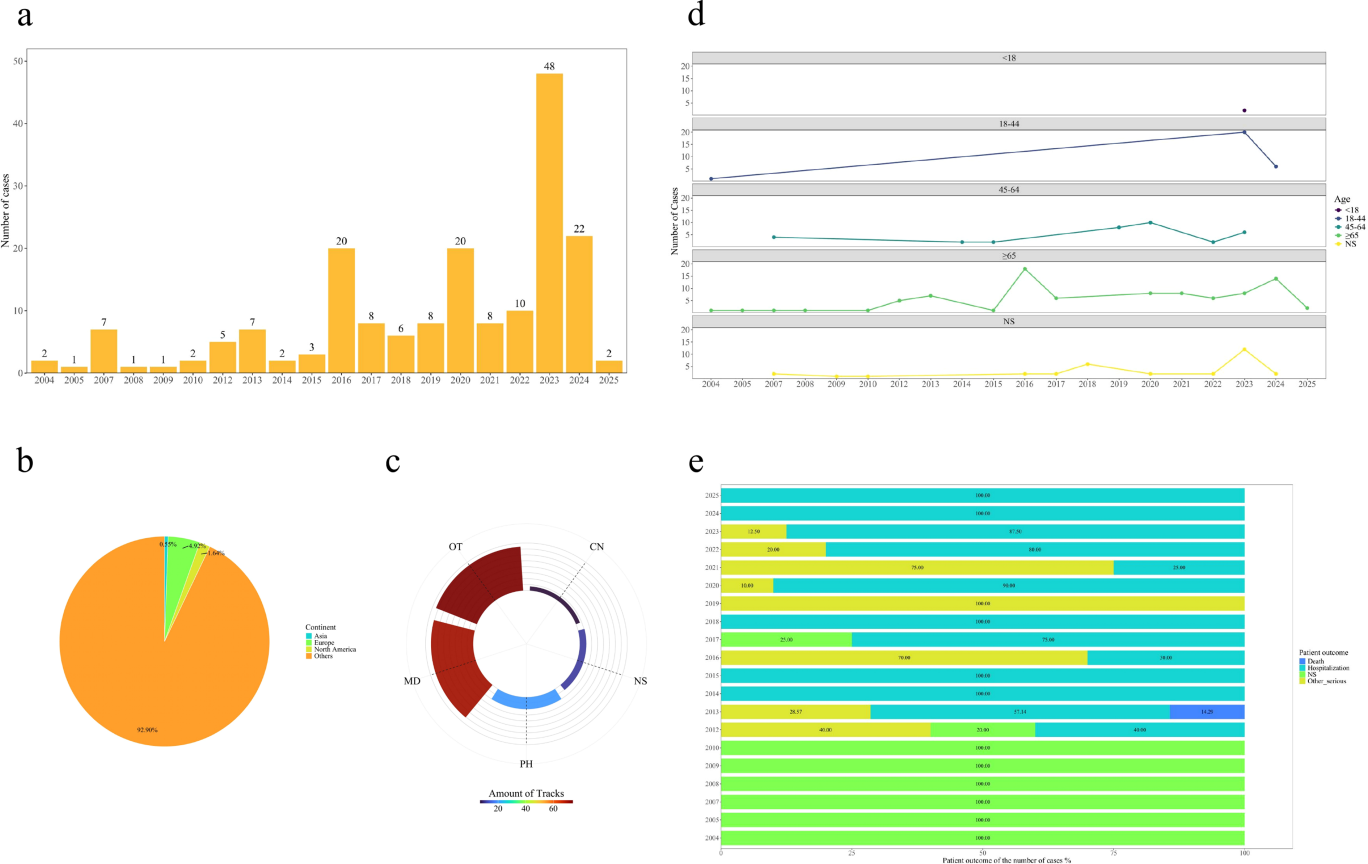
Demographic characteristics of AE reports **(a)** Annual distribution of number of cases; **(b)** Continent of the reporter; **(c)** Occupation of the reporter;**(d)** Age division of the number of cases over time; **(e)** Percentage change in the number of patient outcome reports over time.

The baseline data are presented in **Table 2**. We observed a trend of increasing cases of QAP over the years, with only two cases reported in 2004 and 48 cases in 2023 (**Figure 2a**). Visualizations of key indicators such as age, gender, and patient outcomes are presented in **Figures 2b-d**. The most frequently reported AEs related to pemphigoid were ofloxacin (n=86, 47.0%), followed by ciprofloxacin (n=48, 26.3%), levofloxacin (n=42, 23.0%), moxifloxacin (n=4, 2.2%), and norfloxacin (n=3, 1.6%). Among all QAP cases, females represented the majority (55.7%), compared with males (31.1%). The highest reporting rate was in the ≥65 years age group (48.1%). Of the AE reports, 123 cases (67.2%) required hospitalization, one case (0.5%) resulted in death, and 42 cases (23%) involved other serious medical events (**Figure 2e**).

**Table 2:** Clinical characteristics of five quinolone antibiotics reported in the FAERS database.

Characteristics	Level	Overall (n)
		183
Drugname (%)	CIPROFLOXACIN	48 (26.2)
	LEVOFLOXACIN	42 (23.0)
	MOXIFLOXACIN	4 (2.2)
	NORFLOXACIN	3 (1.6)
	OFLOXACIN	86 (47.0)
Sex (%)	F	102 (55.7)
	M	57 (31.1)
	NS	24 (13.1)
Age (%)	<18	2 (1.1)
	18-44	27 (14.8)
	45-64	34 (18.6)
	≥65	88 (48.1)
	NS	32 (17.5)
Weight (%)	<80	35 (19.1)
	≥80	15 (8.2)
	NS	133 (72.7)
Occupation of the reporter (%)	CN	7 (3.8)
	MD	71 (38.8)
	NS	11 (6.0)
	OT	74 (40.4)
	PH	20 (10.9)
Continent of the reporter (%)	Asia	1 (0.5)
	Europe	9 (4.9)
	North America	3 (1.6)
	Others	170 (92.9)
Outcome (%)	DE	1 (0.5)
	HO	123 (67.2)
	NS	17 (9.3)
	OT	42 (23.0)

### 3.3 Signal strength reported at the PT level

**Figure 3:**
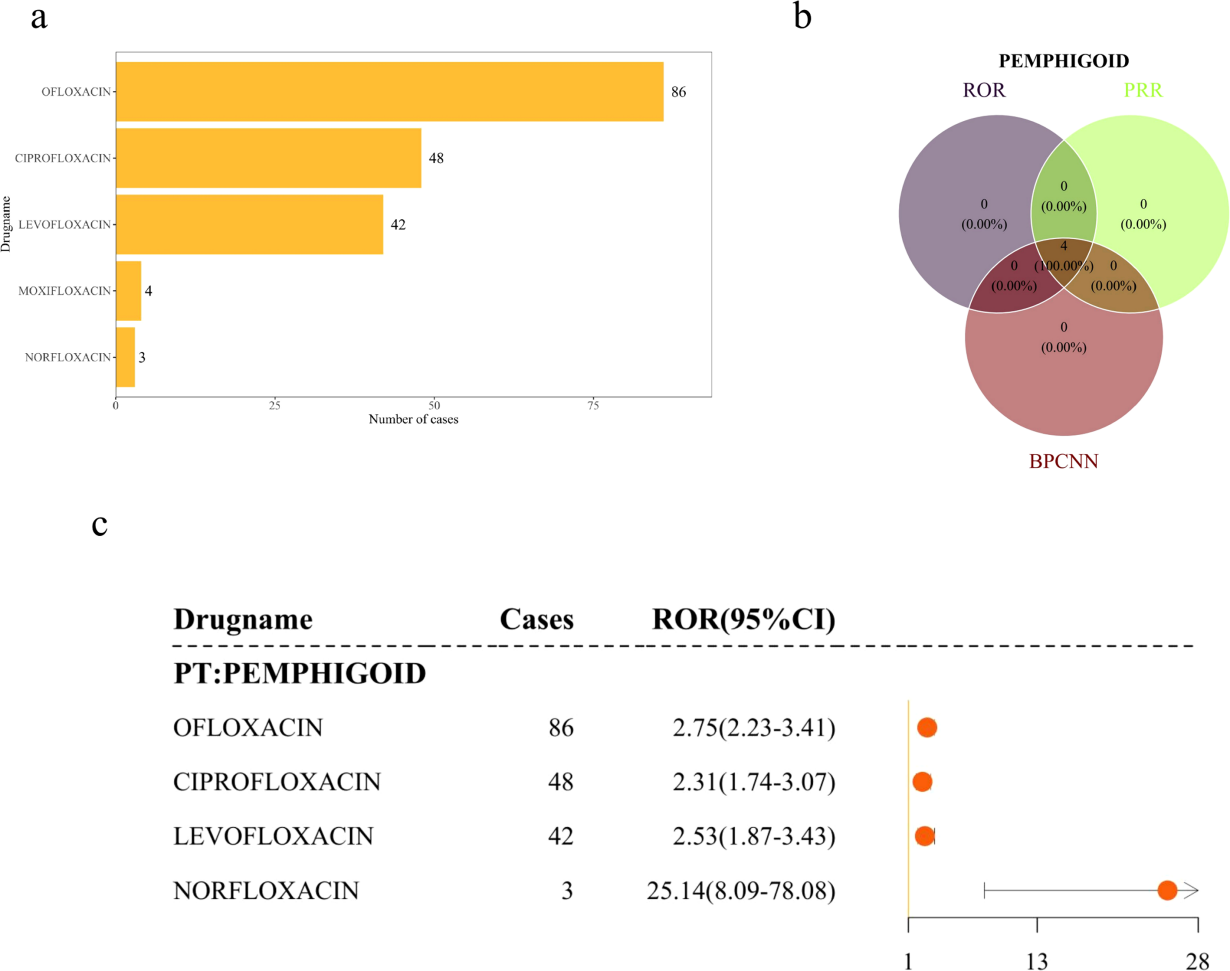
Signal strength of PT-level AEs in the FAERS database. **(a)** Number of PT-level AEs associated with five quinolones; **(b)** Venn diagram depicting quinolones simultaneously meeting signal detection thresholds across all three algorithms (ROR, PRR, BCPNN); **(c)** Forest plot illustrating significant ROR signals for QAP at the PT level.

This study analyzed the association between quinolones and pemphigoid through disproportionality analysis in FAERS. Disproportionality signals were detected for five quinolones, four of which showed significant associations (**Supplementary Table 2**). Ofloxacin, ciprofloxacin, levofloxacin, and norfloxacin all generated positive signals under each of the three disproportionality metrics applied: ROR, PRR, and BCPNN (**Figure 3b**). Among the drugs with robust reporting volumes, ofloxacin exhibited the strongest disproportionality signal for pemphigoid AEs (ROR = 2.75, 95% CI 2.23-3.41), followed by levofloxacin (ROR = 2.53, 95% CI 1.87-3.43) and ciprofloxacin (ROR = 2.31, 95% CI 1.74-3.07). While norfloxacin generated an extraordinarily high numerical value (ROR = 25.14, 95% CI 8.09-78.08), it should be noted that this finding is based on only 3 cases. Due to this extremely small sample size, the statistical stability of this signal is poor and highly susceptible to reporting bias. (**Figure 3c**).

### 3.4 AE subgroup analysis

**Figure 4:**
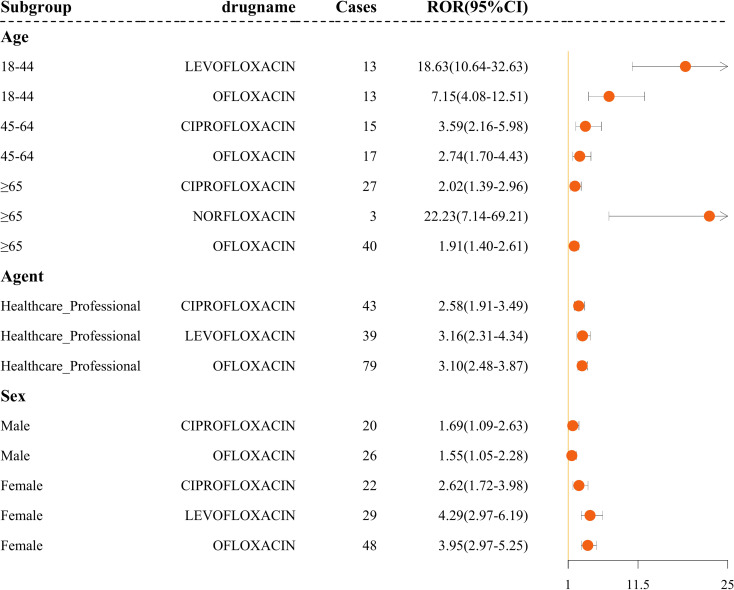
Forest plot of statistically significant ROR signals in subgroup analyses. This chart displays the number of cases and the reporting odds ratio (ROR)with 95%confidence intervals for each subgroup, stratified by age (18-44,45-64,≥65), reporter type (healthcare professionals), and gender (male and female). The chart illustrates subgroup-specific differences in adverse event reporting.

To minimize the confounding effect of demographic factors, we conducted subgroup analyses stratified by age, reporter, and gender. Ofloxacin shows a significant pemphigoid AE signal across all age and gender subgroups. Among the reported cases, 70 involved patients aged 65 or older. Norfloxacin was associated with pemphigoid adverse events exclusively in patients aged ≥65 years (ROR = 22.23), while the signal for levofloxacin reached statistical significance only among females aged 18–44 years. Female patients exposed to ofloxacin and ciprofloxacin had higher reporting odds of pemphigoid AEs than male patients (ROR 2.62 vs. 1.69 for ofloxacin; 3.95 vs. 1.55 for ciprofloxacin). Additionally, reports submitted by healthcare professionals also indicated disproportionality signals for ofloxacin, ciprofloxacin, and levofloxacin (ROR > 2.5 for all) (**Figure 4**).

### 3.5 Timing of AEs

**Figure 5:**
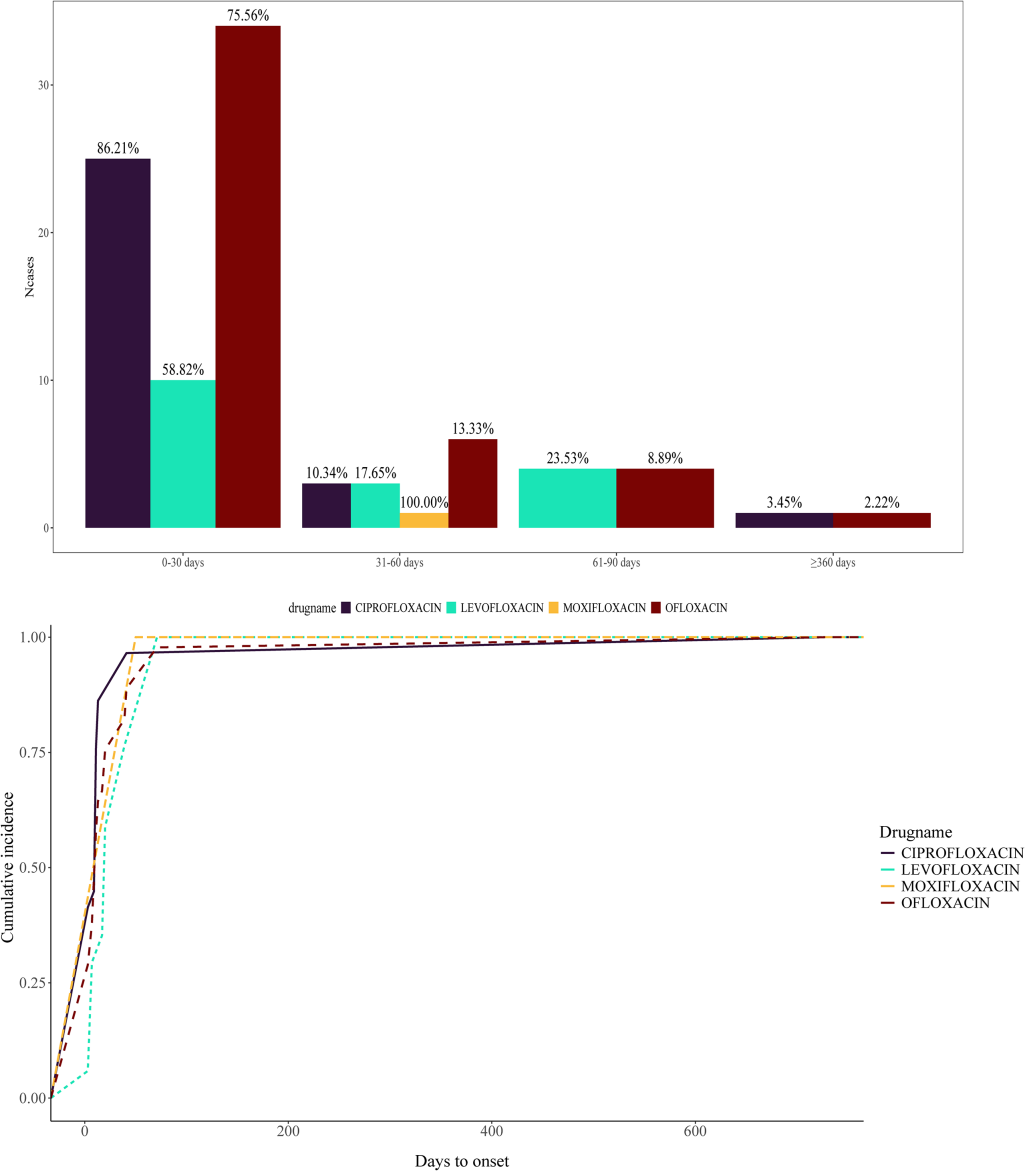
Time of Occurrence of QAP-related AEs. The bar chart shows the distribution of cases by AE induction time interval (0-30, 31-60, 61-90, and ≥360 days). The line graph illustrates the progression of cumulative incidence for each drug.

Reports on AE timing were collected for four quinolones (**Supplementary Table 3**). The pharmacovigilance reports showed that the incubation period for most QAP was within 30 days. For example, all patients treated with levofloxacin developed pemphigoid within 90 days, and 58.82% received a definitive diagnosis within 30 days. Among cases of ciprofloxacin-associated pemphigoid, 86.21% were diagnosed within one month. Additionally, 75.56% of patients with ofloxacin-associated pemphigoid were diagnosed within one month. It was also observed that the onset of AEs associated with quinolones can range from several months to over a year (**Figure 5**). For example, following ciprofloxacin exposure, AEs occurred between 31 and 60 days in 10.34% of cases (n=3) and after one year in 3.45% of cases (n=1).

### 3.6 Interaction analysis between FAERS and literature data

**Figure 6:**
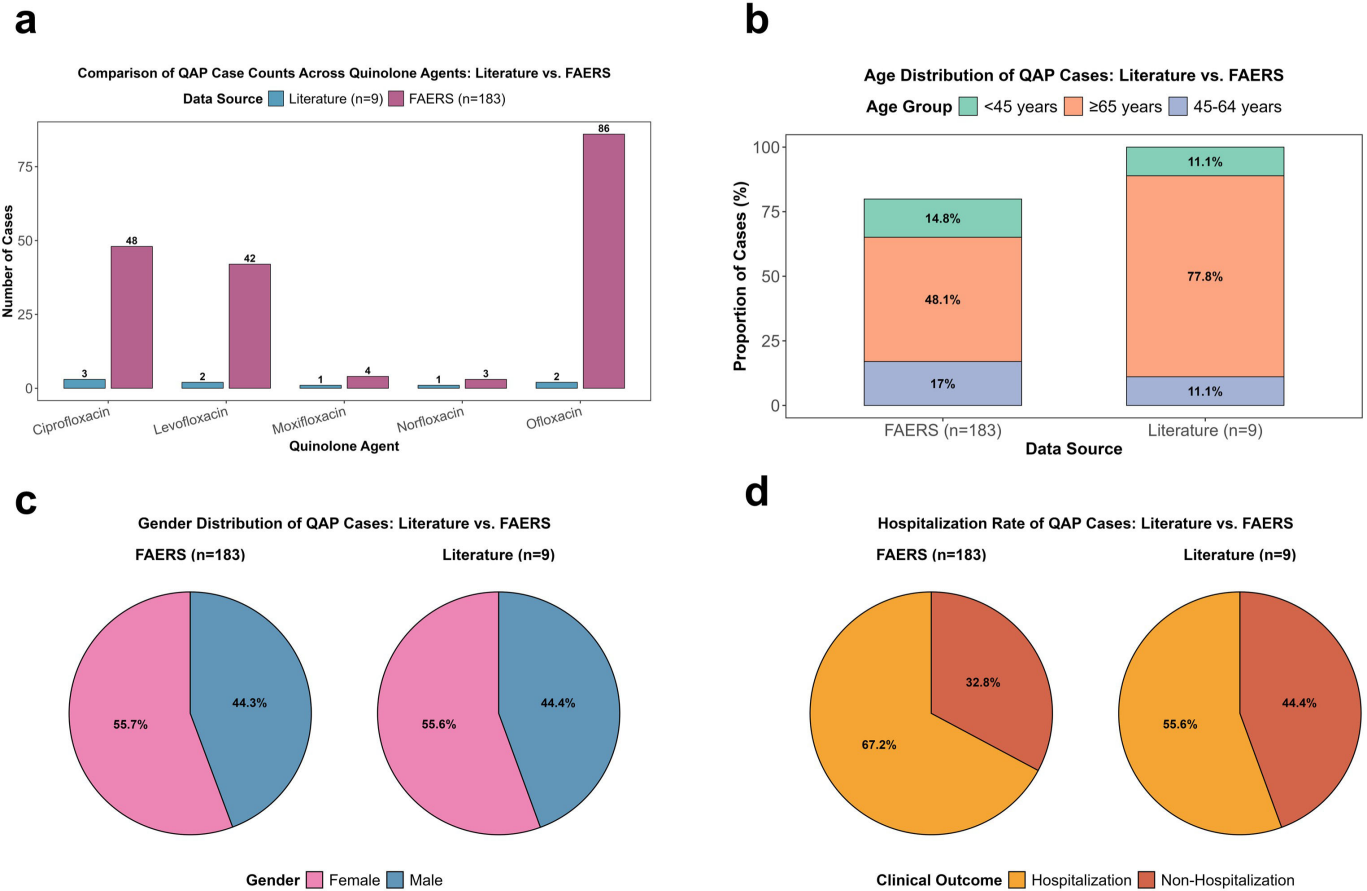
Interaction analysis between FAERS and literature data. **(a)** Consistency of QAP case distribution across quinolone types; **(b)** Consistency of age distribution; **(c)** Consistency of gender distribution; **(d)** Consistency of hospitalization rate.

The interaction analysis utilized the literature as an auxiliary clinical reference to compare core features with the FAERS data. Among 9 literature QAP cases, ciprofloxacin, levofloxacin, and ofloxacin were the most commonly implicated quinolones, together representing 77.8% of cases. Similarly, in FAERS, these three agents comprised 96.2% of all QAP reports and each generated statistically significant ROR signals, corroborating their prominent role in pemphigoid reporting (**Figure 6a**). Both datasets identified the elderly (≥65 years: 77.8% vs 48.1%) as high-risk, with comparable young case proportions (<45 years: 11.1% vs 14.8%) (**Figure 6b**). Females accounted for over half (55.6% vs 55.7%), with stronger ROR signals for ofloxacin and ciprofloxacin in females (**Figure 6c**). Regarding clinical timelines and outcomes, the median latency in the literature was 18 days (range: 4–30 days), aligning with the FAERS finding that most events occurred within 30 days. Hospitalization rates were 55.6% (literature) and 67.2% (FAERS, which included one fatal case), with both sources indicating that most patients recovered after drug withdrawal without long-term sequelae (**Figure 6d**).

## 4 Discussion

Pemphigoid is a group of autoimmune blistering diseases characterized by subepidermal blisters caused by antibody-mediated targeting of structural components of the basement membrane zone (BMZ) (1). Genetic susceptibility, including specific HLA alleles, may predispose individuals to drug-induced pemphigoid, supporting the concept that pharmacological exposures can act as immune triggers rather than direct toxic insults (33). Various medications have been implicated in precipitating pemphigoid through immune activation or antigenic modification of BMZ proteins (34–37), and once initiated, autoimmune responses may persist even after drug withdrawal (18, 38).

Using FAERS-based pharmacovigilance methods, this study identified a significant association between quinolone antibiotics and reports of pemphigoid, which was further supported by interaction analysis with published literature. Strong and consistent safety signals were observed for ofloxacin, levofloxacin, ciprofloxacin, indicating an increased reporting association of pemphigoid associated with these agents.

Antibiotics represent one of the major drug classes implicated in drug-induced pemphigoid (39), however, evidence regarding quinolones has largely been limited to isolated case reports and small case series. By contrast, this study provides population-level real-world evidence that corroborates earlier clinical observations. The predominance of ofloxacin, ciprofloxacin, and levofloxacin in FAERS reports parallels their frequent implication in the literature. Moreover, the consistency between FAERS and published cases in terms of age distribution, female predominance, latency, and clinical outcomes provide a valuable auxiliary reference for the clinical features of these events. While this small literature sample cannot definitively confirm external validity, the observed patterns align with the FAERS data and offer important clinical context regarding the presentation of these adverse drug reactions.

From an immunopharmacological perspective, several features of quinolone-associated pemphigoid support an immune-mediated mechanism. Most cases occurred within 30 days of drug initiation, a latency pattern typical of immune-related adverse drug reactions rather than cumulative toxicity. The high proportion of serious outcomes and hospitalizations further aligns with the clinical severity characteristic of autoimmune blistering diseases.

The underlying biological mechanisms of quinolone-associated pemphigoid remain incompletely defined, but several plausible molecular pathways may explain the observed associations. Existing studies have demonstrated that the core immune mechanism of drug-induced pemphigoid mostly involves the production of autoantibodies targeting the basement membrane zone antigens BP180 (type XVII collagen) and BP230 (19, 20). This process may be triggered by the binding of drugs to host proteins or the alteration of antigen conformation. Quinolone drugs may induce the exposure or modification of epidermal basement membrane antigens through a similar mechanism, thereby promoting the formation of antibody responses. In addition, several studies have suggested that alleles such as HLA-DQB1*03:01 may be associated with susceptibility to drug-related pemphigoid, which is consistent with the genetic background observed in cases related to dipeptidyl peptidase 4 (DPP-4) inhibitors (40, 41). In contrast, DPP-4 inhibitors often exert their pathogenic effects by altering immune regulatory pathways and activating cytokine networks such as interleukin-6 (IL-6) and tumor necrosis factor-α (TNF-α), whereas quinolone drugs may be more inclined to induce autoimmune responses through antigen modification or non-specific immune activation (42). This difference merits further exploration.

Subgroup analyses provided further insight into populations with higher reporting frequencies. Although quinolone-associated pemphigoid was observed across all age groups, elderly individuals (≥65 years) were disproportionately affected, a finding consistent with epidemiological patterns reported for autoimmune blistering diseases, including linear IgA bullous dermatosis (39). Significant ROR signals for ciprofloxacin, norfloxacin, and ofloxacin in older patients underscore the need for cautious prescribing in this population. Although norfloxacin had an extremely high ROR in elderly patients, this estimate was based on only three cases. The small sample size causes substantial statistical instability and makes the estimate prone to outliers and reporting bias. Further validation in large−sample cohort studies is therefore warranted. In contrast, levofloxacin showed a markedly higher reporting signal in females aged 18-44. This susceptibility likely reflects synergistic immunological and pharmacokinetic sex differences. During reproductive years, estrogens act as potent immunostimulants, enhancing B-cell activity and lowering immune tolerance (43). This heightened baseline reactivity facilitates hapten-induced formation of autoantibodies against basement membrane proteins (e.g., BP180) upon levofloxacin exposure (44). Additionally, sex-based differences in drug metabolism may increase relative drug exposure (45). However, since FAERS lacks granular immunogenetic and pharmacokinetic data, this multifaceted hypothesis warrants further validation in controlled studies.

From a clinical perspective, the temporal pattern identified in this study is particularly relevant, as most quinolone-associated pemphigoid events occurred within 30 days of drug initiation. This finding delineates a clinically actionable high-risk window during the first month of therapy, during which close monitoring for blistering manifestations is warranted. The high hospitalization rate and documented fatalities observed in FAERS indicate that quinolone-associated pemphigoid can be clinically severe. Accordingly, careful risk-benefit assessment and early dermatological evaluation should be considered when prescribing ofloxacin, ciprofloxacin, levofloxacin, or norfloxacin, particularly in elderly and female patients.

Several methodological strengths enhance the robustness of these findings. The application of multiple complementary signal detection algorithms reduced method-specific bias. The integration of FAERS data with published literature provided dual-source validation, mitigating limitations inherent to single-database analyses. Finally, the identification of a consistent ~30-day high-risk period offers a practical framework for early monitoring and risk mitigation in clinical practice.

## 5 Limitations

This study has several limitations inherent to analyses based on spontaneous reporting systems. First, FAERS data are subject to reporting bias, underreporting, and variable report quality, which may influence signal strength. Second, although FAERS detects drug-event associations, it cannot establish definitive causality due to unmeasured confounders. A major limitation is our inability to control for polypharmacy and complex comorbidities prevalent in the elderly. Specifically, we did not exclude cases with concomitant high-risk medications, such as DPP-4 inhibitors, aldosterone antagonists or immune checkpoint inhibitors. Furthermore, ‘confounding by indication’ is a critical challenge: since infections can independently trigger autoimmune responses, it is difficult to distinguish whether the pemphigoid was induced by the quinolone or the underlying primary infection. thus, these results represent statistical reporting associations rather than independent causal correlations. Third, incomplete information in some reports, including drug dosage, treatment duration, and detailed clinical characteristics, limited more granular analyses. Fourth, the small number of literature-derived cases included in this study may be subject to publication bias and language bias. Furthermore, published reports predominantly focus on typical or severe cases, which may lead to an underestimation of the proportion of mild or undiagnosed cases. Therefore, the findings of this literature review are mainly used as an auxiliary clinical reference, rather than to confirm the external validity of signals detected in the FAERS database or to draw quantitative inferences regarding the true incidence or risk of quinolone-induced pemphigoid. Future studies incorporating additional data sources, such as prospective cohorts or clinical trial databases, are warranted to further clarify the causal mechanisms underlying quinolone-associated pemphigoid.

## 6 Conclusions

This pharmacovigilance study identified significant reporting associations between quinolone antibiotics and pemphigoid in FAERS. The predominantly early onset of events supports the potential of quinolone-associated pemphigoid as a clinically relevant immune-related adverse drug reaction. Increased clinical vigilance is warranted during the initial period of quinolone therapy, particularly in elderly and female patients.

## Data Availability

Publicly available datasets were analyzed in this study. This data can be found here: https://fis.fda.gov/extensions/FPD-QDE-FAERS/FPD-QDE-FAERS.html.
